# Expression of Kruppel-Like Factor KLF4 in Mouse Hair Follicle Stem Cells Contributes to Cutaneous Wound Healing

**DOI:** 10.1371/journal.pone.0039663

**Published:** 2012-06-20

**Authors:** Juan Li, Hai Zheng, Junfeng Wang, Fang Yu, Rebecca J. Morris, Timothy C. Wang, Shiang Huang, Walden Ai

**Affiliations:** 1 Department of Pathology, Microbiology and Immunology, University of South Carolina School of Medicine, Columbia, South Carolina, United States of America; 2 Centre for Stem Cell Research and Application, Union Hospital, Tongji Medical College, Huazhong University of Science and Technology, Wuhan, China; 3 Department of General Surgery, Union Hospital, Tongji Medical College, Huazhong University of Science and Technology, Wuhan, China; 4 Laboratory of Stem Cells and Cancer, The Hormel Institute, University of Minnesota, Austin, Minnesota, United States of America; 5 Division of Digestive and Liver Diseases, Department of Medicine and Irving Cancer Center, Columbia University, New York, New York, United States of America; University Hospital Hamburg-Eppendorf, Germany

## Abstract

**Background:**

Kruppel-like factor KLF4 is a transcription factor critical for the establishment of the barrier function of the skin. Its function in stem cell biology has been recently recognized. Previous studies have revealed that hair follicle stem cells contribute to cutaneous wound healing. However, expression of KLF4 in hair follicle stem cells and the importance of such expression in cutaneous wound healing have not been investigated.

**Methodology/Principal Findings:**

Quantitative real time polymerase chain reaction (RT-PCR) analysis showed higher KLF4 expression in hair follicle stem cell-enriched mouse skin keratinocytes than that in control keratinocytes. We generated KLF4 promoter-driven enhanced green fluorescence protein (KLF4/EGFP) transgenic mice and tamoxifen-inducible KLF4 knockout mice by crossing KLF4 promoter-driven Cre recombinase fused with tamoxifen-inducible estrogen receptor (KLF4/CreER™) transgenic mice with KLF4(flox) mice. KLF4/EGFP cells purified from dorsal skin keratinocytes of KLF4/EGFP transgenic mice were co-localized with 5-bromo-2'-deoxyuridine (BrdU)-label retaining cells by flow cytometric analysis and immunohistochemistry. Lineage tracing was performed in the context of cutaneous wound healing, using KLF4/CreER™ and Rosa26RLacZ double transgenic mice, to examine the involvement of KLF4 in wound healing. We found that KLF4 expressing cells were likely derived from bulge stem cells. In addition, KLF4 expressing multipotent cells migrated to the wound and contributed to the wound healing. After knocking out KLF4 by tamoxifen induction of KLF4/CreER™ and KLF4(flox) double transgenic mice, we found that the population of bulge stem cell-enriched population was decreased, which was accompanied by significantly delayed cutaneous wound healing. Consistently, KLF4 knockdown by KLF4-specific small hairpin RNA in human A431 epidermoid carcinoma cells decreased the stem cell population and was accompanied by compromised cell migration.

**Conclusions/Significance:**

KLF4 expression in mouse hair bulge stem cells plays an important role in cutaneous wound healing. These findings may enable future development of KLF4-based therapeutic strategies aimed at accelerating cutaneous wound closure.

## Introduction

Skin is a continuously regenerating organ composed of a basal layer of proliferating cells and suprabasal layers of terminally differentiating cells that transit toward and are sloughed from skin surface [1]. Epidermal renewal is thought to be controlled by stem cells located either in the basal layer of the interfollicular epidermis (IFE) or in the deepest portion of permanent hair follicle called bulge [2]. Mouse hair follicle stem cells which reside in the hair follicle bulge are characterized by expression of CD34 cell-surface marker [3], [4], [5], retention of either DNA or histone labels over long periods [6], [7], and expression of Leucine-rich repeats and immunoglobin-like domain protein 1 (Lrig1) [8], [9]. It has been shown that expression of CD49f, which is also known as α6 integrin, was continuous throughout the basal layer of IFE and hair follicles [10]. Wound healing is an important response of skin that repairs itself after injury. Regeneration of epidermis after wounding involves activation, migration and proliferation of keratinocytes from both the surrounding epidermis and the adnexal structures such as hair follicles [11], [12], [13]. The discovery of properties of epidermal stem cells led to the hypothesis that these stem cells play a critical role in epidermal repair after wounding. Previous work has reported that bulge stem cells rapidly respond to wounding and migrate towards the IFE to help with the rapid hair-follicle regeneration and that bulge-derived cells are transient amplifying cells committed to differentiation [9], [12], [14]. However, the role and contribution of keratinocytes derived from hair follicle bulge stem cells to cutaneous wound healing need further elucidation.

Kruppel like factor 4 (KLF4) is a transcriptional factor previously known as gut-enriched Kruppel-like factor. As a member of Kruppel-like factor family, KLF4 is highly expressed in the gastrointestinal tract and other epithelial tissues including skin [15], [16]. KLF4 has been thoroughly investigated with respect to its role in cell cycle arrest and cellular differentiation [17], [18], [19], [20], [21]. Previous work has shown that KLF4 is required for establishing the barrier function of skin. KLF4 null mice die shortly after birth due to loss of skin barrier function without morphological and biochemical alterations. Instead, knockout of KLF4 selectively perturbs the differentiation of late-stage structures, including the cornified envelope [1]. Recently, KLF4 has been shown to have key functions in stem cell biology. Gene profiling results showed that KLF4 expression was elevated in mammary gland stem cells [22] and in hematopoietic stem cells [23], [24], [25]. Forced expression of KLF4 was found to inhibit embryonic stem cell differentiation and increase their self-renewal capacity [26], [27]. In addition, KLF4, together with three other transcription factors, Oct4, Sox2, and c-Myc, transformed mouse embryonic and adult fibroblasts into induced pluripotent stem cells [28]. We recently found that KLF4 is required for the maintenance of breast cancer stem cells [29]. These observations suggest that KLF4 plays an indispensable role in the maintenance of stem cells. However, expression of KLF4 in epidermal stem cells and its potential function in cutaneous wound healing have not been investigated.

In this study, we showed that KLF4 is likely expressed in mouse epidermal stem cells. A decreased number of hair bulge stem cells was observed in KLF4 knockout mice, which was accompanied by decreased ability of colony formation from these cells when compared to those from control mice, suggesting that KLF4 may be required for the maintenance of skin hair follicle stem cells. Notably, KLF4 deficiency delayed the process of mouse cutaneous wound healing, during which process KLF4-expressing multipotent cells migrated towards the wound area.

## Materials and Methods

### Ethics statement

The study has been approved by the Institutional Biosafety Committee (IBC) and by the Institutional Animal Care and Use Committees (IACUC) of University of South Carolina (Proposal number 1867).

### Purification of mouse dorsal skin keratinocytes, flow cytometry, and cell sorting

Purification of mouse dorsal skin keratinocytes was performed as reported previously [30], [31]. They were collected and resuspended at 1 million cells per ml in PBS with 0.5% BSA and then stained with PE-conjugated antibody to CD49f, APC-conjugated antibody to Lrig1, FITC or Percp-Cy5.5-conjugated antibody to CD34 (eBioscience) for 1 h on ice. Staining analysis was carried out by using a flow cytometer (Cytomics FC 500; Beckman Coulter). Cell sorting was performed using the FACSAria cell sorter (BD Biosciences).

### RNA extraction and quantitative RT-PCR analysis

Total RNA was prepared using Trizol Reagent (Invitrogen) according to the manufacturer's instructions. First-strand cDNA synthesis was performed using Oligo (dT) and Superscript III reverse transcriptase (Invitrogen). Real-time PCR was performed as described previously [32]. Quantitative real-time PCR primer sequences were listed in Table 1.

**Table 1:** Primers for quantitative RT-PCR.

	Sense	Antisense
KLF4	5′-GAAATTCGCCCGCTCCGATGA-3′	5′-CTGTGTGTTTGCGGTAGTGCC-3′
GAPDH	5′-TTCGACAGTCAGCCGCATCTTCTT-3′	5′-CAGGCGCCCAAYACGACCAAATC-3′
CD34	5′-TGGGTCAAGTTGTGGTGGGAA-3′	5′-GAAGAGGCGAGAGAGGAGAAATG-3′
FEX	5′-CACCGCTTGTATCATTTGTTCAGC-3′	5′-CATCCAACGAGTCTTCTCACTATG-3′
Cktsfb1	5′-TCCCATAGCCCATCCCTTTC-3′	5′-TCTGTCCCGTTTGCCATCAC-3′
DKK 3	5′-TACCTCTGAAAGCCAGTGCTCG-3′	5′-CTTGGTTGTGACTTCTCGGTGTG-3′
Bmp4	5′-GATTCCTGGTAACCGAATGC-3′	5′-GCAGCCCAAACATCTGTAGA-3′
KLF5	5′-GAGCTGGTCCAGACAAGATG-3′	5′-TGTCTTGATCTGTGTTACGC-3′

### Generation of the KLF4/EGFP transgenic mouse model

PKD4-NICD-EGFP plasmid (a kind gift from Dr. Andrew Leiter at University of Massachusetts) was used as PCR templates to amplify fragments containing EGFP. Bacterial artificial chromosome (BAC) clone # RP23-322L22 was obtained from Children's Hospital Oakland Research Institute (CHORI) and the bac clone was then introduced into a bacterial strain BL250 (kindly provided by Dr. Neal G. Copeland at NIH). BL250 bacteria containing RP23-322L22 were made competent and transformed with the amplified EGFP fragment by electroporation according to a previous report [33]. Clones in which the amplified fragment was inserted via homologous recombination were selected using resistance to chloramphenicol and kanamycin. Selected colonies were screened for correct recombination by amplification using EGFP- specific primers. The FRT-flanked kanamycin resistant (KANA) gene was excised from KLF4/EGFP-KANA BAC clones by expressing the FLP recombinase. A diagram of the construction strategy to generate a KLF4/EGFP transgene is shown in Figure S1A. Clamped homogenous electric fields (CHEF) gel electrophoresis and DNA sequencing confirmed correct transgene constructions and integrity of the BAC flanking sequence. The KLF4/EGFP BAC was then purified for pronuclear microinjection. Potential founder mice (B6/CBA mixed background) were genotyped by tail DNA amplification using primers specific for the EGFP coding sequence. The founder transgenic mice were then backcrossed 6–8 generations to obtain the pure C57BL/6 background. The KLF4/CreER™ transgenic model using a construct containing Cre recombinase cDNA fused with a tamoxifen-inducible (TM) estrogen receptor (ER) gene (a plasmid kindly provided by Dr. Douglas Melton at Harvard Medical School) as the transgene was similarly generated [34]. KLF4 (flox+/+) mouse model was generated as described [35].

### Immunohistochemistry (IHC)

Following sacrifice, skin tissues were dissected, embedded in paraffin and sectioned at 4 µm. IHC was performed using a standard protocol. Signal was detected using the KPL's 3,3-Diaminobenzidine (DAB) Reagent Set (KPL, Gaithersburg, MD) according to the manufacturer's instructions, and counterstaining was carried out with Mayer hematoxylin. The following primary antibodies were used: rabbit anti-KLF4 monoclonal antibody (1∶500, Genespin, Milano, Italy), rabbit anti-Ki67 antibody (1∶500, Abcam), rat anti-BrdU antibody (1∶100, AbD serotec, Oxford, UK).

### X-gal staining of KLF4/CreER™ and Rosa26RLac Z double transgenic mice

Freshly obtained mouse dorsal skin with or without wound placement were fixed with 4% paraformaldehyde for 6 hours followed by 30% sucrose overnight. The samples were embedded in OCT and sectioned at 10 µm. To detect β-Galactosidase activity, frozen sections were washed 3 times for 5 min with rinse buffer (2 mM MgCl_2_/0.1% NP40/PBS) and stained for 24 h in a solution consisting of 1 mg/ml X-gal, 5 mM ferrothiocyanide and 5 mM ferrithiocyanide in rinse buffer. The slides were stained with fast red with or without xylene treatment before being photographed under a microscope.

### Colony formation assay

Primary keratinocytes purified from mouse dorsal skin were seeded at 2×10^3^ cells per 60-mm dish on 3T3 feeder layer. The growth medium was changed every other day. Sixteen days later, cells were fixed with PBS-buffered 4% formaldehyde and stained with 0.5% rhodamine B (Sigma) to visualize the keratinocyte colonies. Three independent experiments were conducted and analyzed.

### BrdU labeling, induction of KLF4 knockout and placement of mouse dorsal wounds

Mice were housed in micro-isolator; solid-bottomed polycarbonate cages under a pathogen free condition and experiments were approved by the Institutional Animal Care and Use Committees of University of South Carolina. Three-day-old KLF4/EGFP mice were injected intraperitoneally with BrdU (75mg/kg) once a day for five consecutive days. Mice were maintained for three months after final BrdU injection to trace LRC. Cre recombinase was induced by daily intraperitoneal injection of tamoxifen (100mg/kg) or topical application of 4-hydroxyl-tamoxifen (1mg/0.2ml acetone) (Sigma, St Louis, MO) for 5 consecutive days. *In vivo* wound healing study was performed after indicated induction. The backs of the mice were shaved and sterilized with 1% iodine solution followed by alcohol. Two full thickness wounds in parallel were made using a dermal biopsy punch of 8mm or 5mm in diameter [36]. After wound placement, mice were housed separately and wounds were observed and photographed on days 1, 5, and 10. The wound closure was determined by the convergence of circular skin edges after wound placement.

### Generation of KLF4 knockdown and overexpression stable cell lines

Human epidermoid carcinoma cell line A431 cells were purchased from ATCC and cultured in Dulbecco's modified Eagle's medium (Invitrogen) supplemented with 10% fetal bovine serum, 100 U/ml penicillin, 100 μg/ml streptomycin, and 2 mM L-glutamine at 37°C in a humidified atmosphere of 5% CO_2_. To generate stable cell lines, plasmids containing KLF4 specific shRNA (pRetroSuper-KLF4#2, designated as shKLF4) or the control vector (pRetroSuper, designated as siCon) [37] and KLF4 overexpression vector (pBabePuro-DN KLF4, designated as KLF4-N) and the control vector (pBabePuro, designated as Con) kindly provided by Daniel S. Peeper at Netherlands Cancer Institute, and were transfected into A431 cells using Lipofectamine^TM^ 2000. 48 h after transfection, cells were trypsinized and reseeded into 10-cm culture plates. Cells were selected with puromycin at a final concentration of 2μg/ml for 3 weeks. Single colonies were selected, confirmed, and expanded for the subsequent experiments.

### Protein extraction and Western Blotting analysis

Total proteins from culture cells were prepared using radio-immunoprecipitation assay (RIPA) buffer, and 50µg of proteins were separated by 12% SDS-PAGE gel followed by Western blotting analysis as previously described [32]. The antibodies used were: rabbit monoclonal anti-KLF4 (1∶1500), anti-p21 (1∶1000), and anti-α-Tubulin (1∶4000, Santa Cruz Biotechnology).

### In vitro scratch assay

A431 cells were cultured to full confluence, and then the medium was changed to DMEM with 0.5% FBS 2h before scratching. The scratch was performed by a 200 µl pipette tip. The detached cells were removed and the remaining cells incubated in DMEM with 0.5% FBS [38]. 12 h later, plates were imaged under Nikon microscope using a 10× Phase contrast objective.

### Statistical analysis

Two-way ANOVA and two-tailed *t*-tests were employed to analyze data using Prism 5.0 software (GraphPad). *P<*0.05 was defined as statistically significant.

## Results

### Expression of KLF4 in mouse hair follicle stem cells

**Figure 1:**
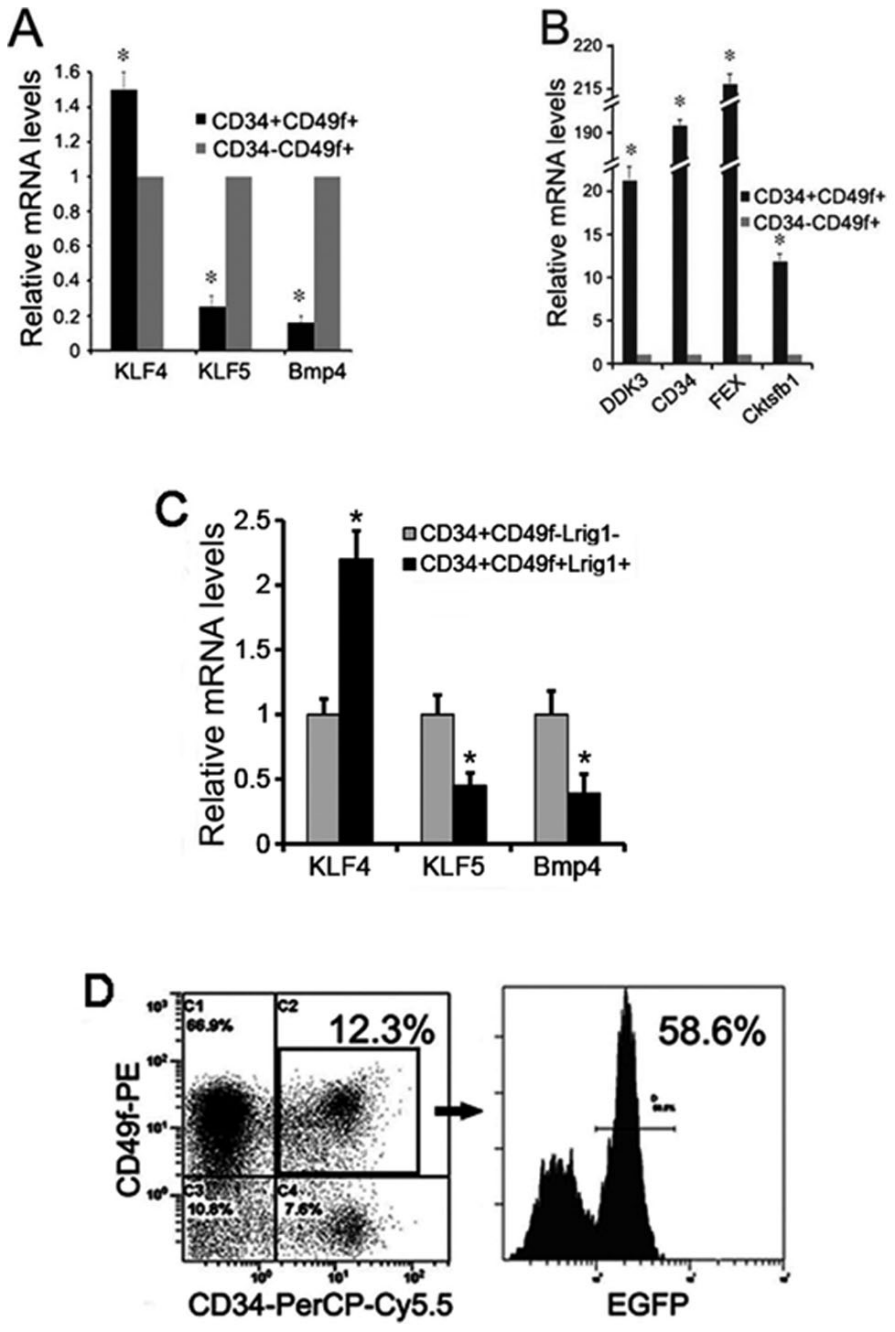
Expression of KLF4 in mouse hair follicle stem cells. (A to C) Quantitative RT-PCR was performed to detect the expression of KLF4, differentiation markers KLF5 and Bmp4 (A, C), and stem cells markers CD34, FEX, Cktsfb1 and DDK3 (B) in different populations isolated by FACS using anti-CD34, anti-CD49f, and anti-Lrig1 antibodies in wild type mice, a procedure that was also described in D. 1×106 sorted cells in each population were used. Values were expressed as mean ± SM of three independent experiments. *P<0.05 vs. control. (D) The proportion of CD34+/CD49f+ cells (left) purified from 6-week-old KLF4/EGFP mice was examined by flow cytometry. KLF4/EGFP-expressing cells in CD34+/CD49f+ population were shown in the right panel. Values in D were expressed as an average of three independent experiments.

To examine if KLF4 is enriched in mouse epidermal stem cells, we first purified these cells by fluorescent activated cell sorting from the skin of 6-week-old wild-type C57BL/6 mice and performed quantitative RT-PCR analysis. Antibodies against CD34 and CD49f, two markers of skin bulge stem cells, were used to enrich mouse skin bulge stem cells (CD34+/CD49f+ cells). CD34, G protein coupled receptor expressed in follicles (FEX) [39], gremlin, cysteine knot superfamily 1, BMP antagonist 1 (Cktsfb1), and dickkopf 3 (DDK3), highly expressed in CD34 enriched mouse keratinocyte stem cells, and KLF5 and bone morphogenesis protein 4 (Bmp4), highly expressed in differentiated cells [4], were used as controls. As expected, significantly increased expression of CD34, FEX, Cktsfb1 and DDK3 and decreased expression of KLF5 and Bmp4 were found in CD34+/CD49f+ population when compared to those in CD34-/CD49f+ cells (Figure 1A, B). Importantly, KLF4 expression showed a statistically significant increase in CD34+/CD49f+ population (51.5% increase) compared to that in CD34-/CD49f+ cells. Additional enrichment of bulge stem cells using CD34, CD49f, and Lrig1 antibodies [9] revealed a more robust KLF4 expression (>2 fold increase) in CD34+CD49f+Lrig1+ population when compared to that in CD34+CD49f-Lrig1- cells (Figure 1C). To further examine KLF4 expression in mouse hair follicle stem cells *in vivo*, we generated a KLF4 promoter-driven EGFP (KLF4/EGFP) transgenic mouse model (Figure S1). Flow cytometric analysis showed that 12.3% of purified mouse dorsal keratinocytes expressed both CD34 and CD49f (Figure 1D left), about 58% of which were EGFP-positive (Figure 1D right).

### KLF4-expressing cells possessed a label retaining property

**Figure 2:**
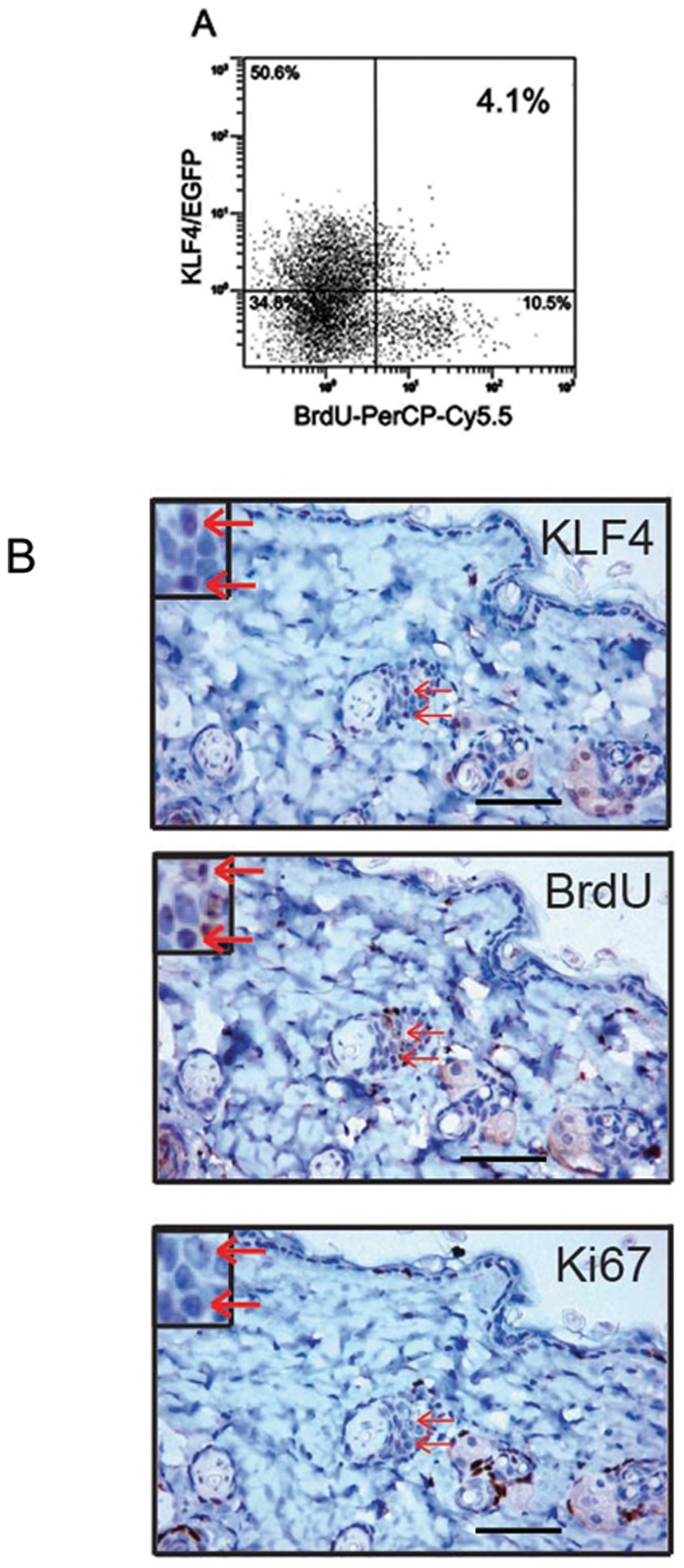
KLF4-expressing cells possessed label retaining property. (A and B) 3-day-old KLF4/EGFP mice were injected with BrdU (75mg/kg) for 5 consecutive days. BrdU-positive cells were examined 3 months later by flow cytometry (A) and immunohistochemical staining (B). Values of A were expressed as an average of three independent experiments and results of B are representative of three separate experiments. Anti-KLF4, anti-BrdU, and anti-Ki67 antibodies were used to stain consecutive slides in B. Insets show enlarged portion of the staining indicating co-localization of KLF4 and BrdU positive cells with no Ki67 signals (red arrows). Scale bars, 50 µm.

The label retention cell assay, a viable method used to locate stem cells and to identify them as slow dividing, quiescent or segregating chromosomes asymmetrically [40], was performed *in vivo*. Three-day-old KLF4/EGFP mice were injected with BrdU and left for an extended period (12 weeks) to label epidermal stem cells as label-retaining cells (LRCs) compared with transit amplifying cells in which the label was diluted after a prolonged chase[41]. As shown in Figure 2A, 14.6% BrdU-positive cells were observed in purified keratinocytes, and 4.1% of them were both BrdU and KLF4/EGFP positive cells. The quiescent nature of these cells was confirmed by lack of proliferation by immunohistochemistry. KLF4 staining was colocalized with that of BrdU, while proliferation was absent as indicted by negative staining of Ki67, a proliferation marker (Figure 2B, red arrows).

### Lineage tracing to show possible KLF4 expression in mouse epidermal multipotent cells

**Figure 3:**
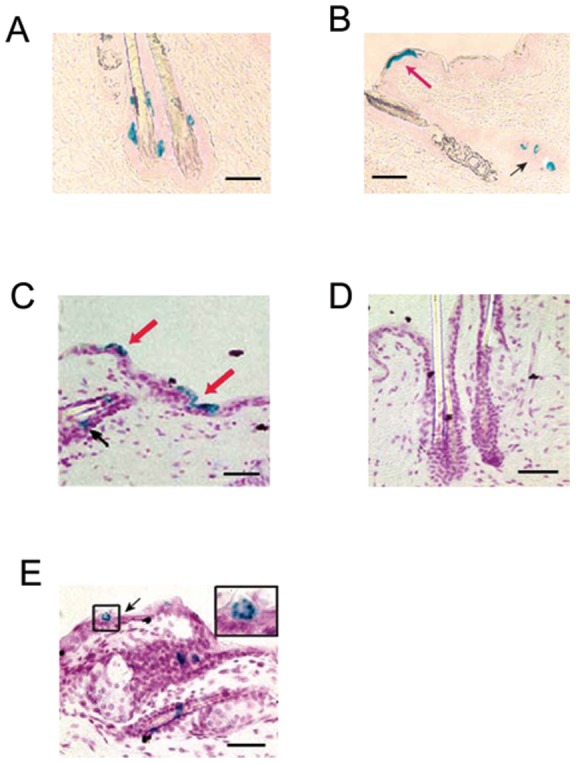
KLF4-expressing hair follicle stem cells were examined by lineage tracing. KLF4/CreER™/Rosa26RLacZ mice were induced by tamoxifen (100mg/kg) for 5 consecutive days at 6-week-old. 4 weeks later X-gal staining was performed. Potential KLF4 expression in interfollicular epidermis (shown by red arrows in B, C) and bulge area (A, and black arrows in B, C) was shown. A typical epithelial proliferation unit was shown in E (inset). Control staining was shown in D using KLF4/CreER™/Rosa26RLacZ mice with mock induction. Representative images were shown from 5 mice in each treatment group. Note that fixation was performed without xylene in A and B. Scale bars, 80 µm.

Next we performed lineage tracing experiments, the most convincing *in vivo* approach to demonstrate gene expression in stem cells, using our tamoxifen-inducible KLF4 promoter-driven Cre recombinase (KLF4/CreER™) transgenic mice [34] (Figure S1). After tamoxifen induction, KLF4-expressing cells and their progenies were permanently labeled with β-galactosidase (LacZ) and turned blue after X-gal staining in KLF4/CreER™ and Rosa26RLacZ double transgenic mice. 4 weeks after tamoxifen induction, LacZ-positive cells were observed in the possible bulge area (Figure 3A and black arrows in Figure 3B, C) and IFE (Figure 3B, C, red arrows). This observation was consistent with previously proposed models regarding the position of stem and progenitor cells [42], [43]. The quiescent nature of possible bulge stem cells was also shown by limited blue cells in this setting (Figure S2). No blue cells were detected when KLF4/CreER™/Rosa26RLacZ double transgenic mice were treated with vehicle control or wild-type mice treated with tamoxifen (Figure 3D and data not shown). Presence of blue cells in a typical structure of epidermal proliferation unit (Figure 3E) also suggests a multipotent and clonal nature of KLF4 expressing cells [44].

### Knockout of KLF4 decreased stem cell population and self-renewal potential

**Figure 4:**
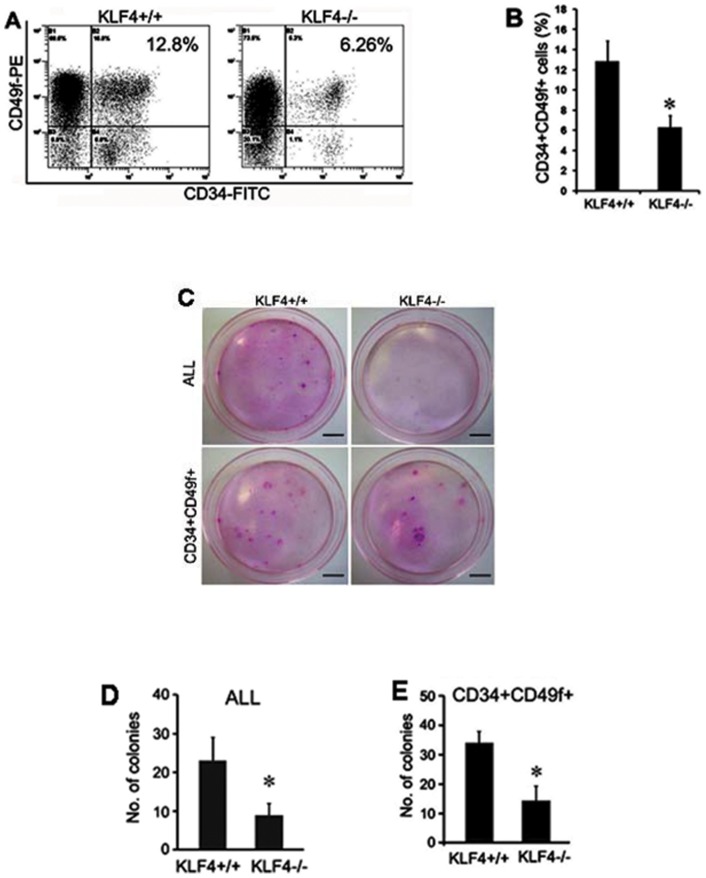
Knockout of KLF4 decreased hair follicle stem cell population and self-renewal potential. (A) Dorsal skin keratinocytes isolated from control (KLF4+/+) and KLF4 knockout (KLF4−/−) mice were analyzed by flow cytometry using mouse epidermal stem cell markers CD34 and CD49f. B. Quantification of data from A was presented. Values were expressed as mean ± SM of three independent experiments. *P<0.05 vs. control. (C) Colony formation assay was performed using total dorsal skin keratinocytes (top) or sorted CD34+/CD49f+ cells (bottom) from control (left) and KLF4 knockout (right) mice. Typical images were shown from three separate experiments. (D and E) Quantitation of the colony numbers from 2000 seeded keratinocytes in (C). Data shown were the mean ± SM of three separate experiments. *P<0.05 vs. control. Scale bars, 1 cm.

To evaluate the effect of KLF4 on mouse epidermal stem cells *in vivo*, two stem cell markers CD34 and CD49f were used to detect and sort keratinocytes from 6–8 week-old control (KLF4+/+) and KLF4 knockout (KLF4−/−) mice generated by induction of KLF4/CreER™(+/−) and KLF4(flox+/+) double transgenic mice with tamoxifen. While no obvious phenotypic changes of mouse skin or hairs were observed eight weeks after tamoxifen induction, we found that the number and the proliferation of hair follicles were increased 45]. As shown in Figure 4A and 4B, population of CD34+/CD49f+ cells decreased from 12.8%±1.96% to 6.26%±1.12% upon KLF4 knockout. To test whether KLF4 knockout is associated with any defects of self-renewal of stem cells, we used purified mouse dorsal skin keratinocytes to perform colony-formation assay. As shown in Figure 4C and D, 22 colonies were formed from 2000 wild type keratinocytes. The colony number decreased to 9 from 2000 KLF4 knockout keratinocytes (*P*<0.05, *n* = 3). This trend hold true when the same number of sorted CD34+/CD49f+ cells was used for the same assay (Figure 4C, E, *P*<0.05, *n* = 3).

### KLF4-expressing multipotent stem cells contributed to acute wound healing

**Figure 5:**
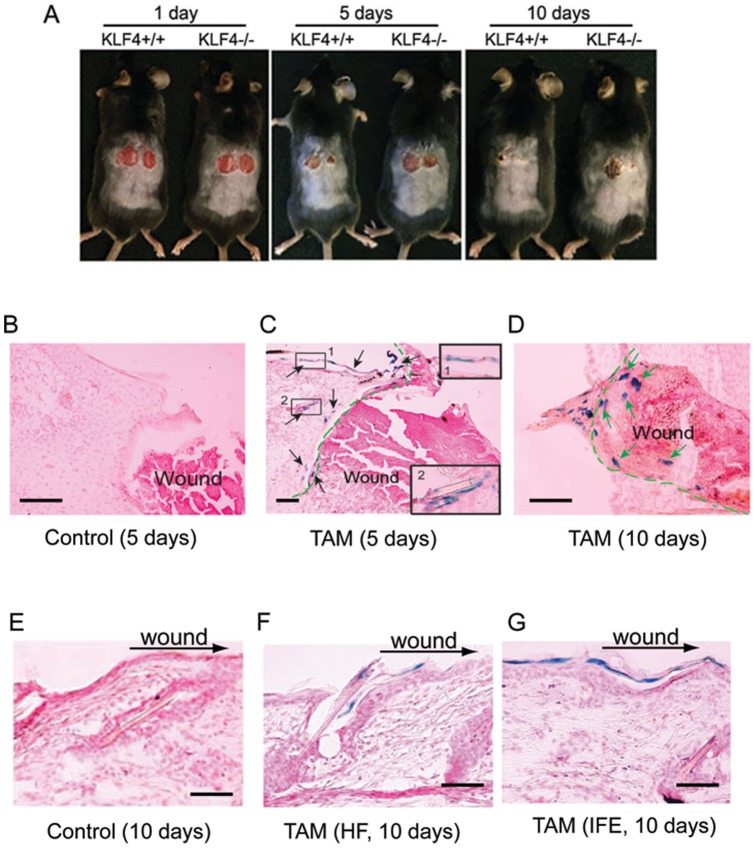
KLF4-expressing multipotent cells contributed to cutaneous wound healing. (A) KLF4 knockout was induced as described in Material and Methods. Pictures were taken 1, 5, and 10 days after two parallel 8mm wounds were placed into backs of the mice. Note that the left wounds were introduced first, and they appeared severer than the right ones because of physical stretch. (B to G) 5mm wounds were introduced into the backs of KLF4/CreER™/Rosa26RLacZ mice 5 (B, C) or 10 days (D–G) after using control (B, E) or tamoxifen (C, D, F, G) induction and X-gal staining was performed. Blue strips on epidermis were shown in C (inset 1) and G. Blue cells was indicated by black arrows outside (C) and by green arrows inside (D) the conjunction of the wound (separated by dashed green lines). Inset 2 in C showed blue cells around hair follicles. Migration of KLF4 expressing multipotent cells from hair follicles (F) and interfollicular epidermis towards the wound area was detected similarly. Results shown are representative of three independent experiments. Scale bars, 80 µm.

KLF4 is critical for skin barrier formation [1]. To test the hypothesis that KLF4 is important in maintaining skin integrity, two full-thickness wounds in parallel were made in KLF4/Cre(+/−)/KLF4(flox+/+) mice. As shown in Figure 5A, wounds started to heal within 5 days in control mice (KLF4+/+). Wound closure was observed 10 days after wound placement. However, in KLF4 knockout mice (KLF4−/−), the healing process was significantly delayed. To test the contribution of KLF4-expressing stem cells to wound healing, lineage tracing was performed using KLF4/CreER™/Rosa26RLacZ mice after tamoxifen induction. When compared to the control (Figure 5B), 5 days after wound placement, blue cells as indicated by black arrows were observed in superbasal layer (inset 1) and around hair follicles (inset 2) in the proximity of but outside the wound (Figure 5C). Ten days after the wound placement, blue cells migrated inside the junction of the wound and the adjacent normal tissue (green arrows in Figure 5D). In addition, activation of KLF4-expressing epidermal multipotent cells was shown by detection of blue cells upon wound placement 3 months and 8 months after tamoxifen induction of KLF4/CreER™/Rosa26RLacZ mice (Figure S3). Furthermore, when compared to the control (Figure 5E), it appears that migration of blue cells towards the wound occurred from both hair follicles (Figure 5F) and from epidermal suprabasal layer (Figure 5G).

### Knockdown of KLF4 decreased the population of cancer stem cells and cell migration in A431 cells

**Figure 6:**
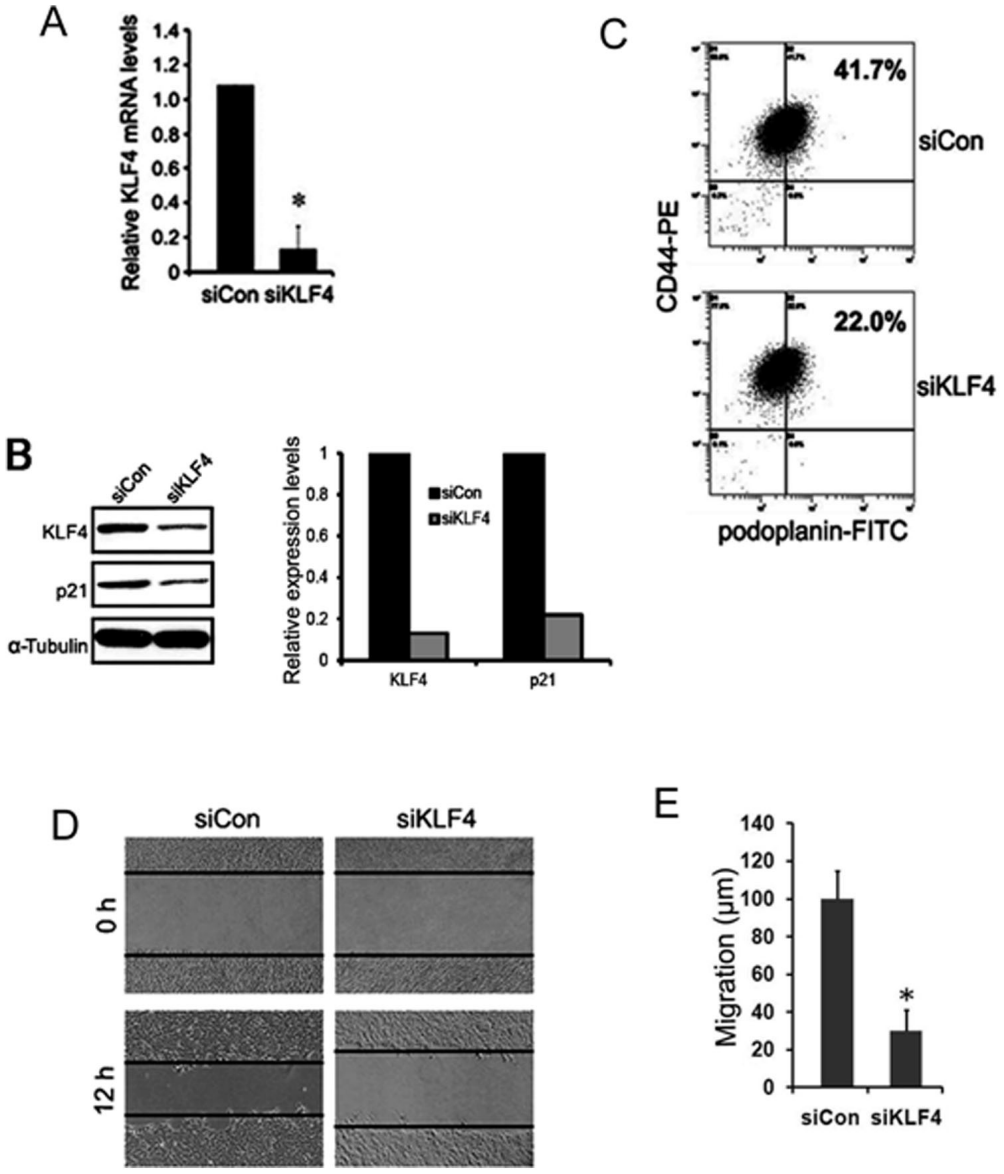
KLF4 knockdown decreased the population of cancer stem cells and cell migration in A431 cells. (A) KLF4 expression was examined in control (siCon) and KLF4 knockdown (siKLF4) cells by real time PCR. *P<0.05 vs. control. (B) Down regulation of KLF4 was accompanied by reduced expression of its downstream target p21 by Western Blotting analysis (Left) and the corresponding densitometry quantification (Right). (C) Flow cytometric analysis of cancer stem cells using CD44 and podoplanin as the markers in siCon and siKLF4 cells. Values were expressed as an average of three independent experiments. (D) Scratch assays were performed using siCon and siKLF4 cells. 2×10^6^ cells were cultured to confluence, then scratched and photographed immediately (0 h) or after 12 hours (12 h). (E) Quantification of cell migration during 12 h after scratching. Error bars represent standard error from three separate experiments. *P<0.05 vs. control.

To further test the effect of KLF4 on maintenance of stem cells and on stem cell-related cell migration, we first generated KLF4 knockdown stable cells by transduction with an established KLF4 shRNA, which specifically knocked down KLF4 with a high efficiency [37], in A431 epidermoid carcinoma cells. Successful KLF4 knockdown was shown by decreased mRNA and protein levels of KLF4 and p21, one of KLF4 target genes, when compared to that in control cells (Figure 6A, B). Previous work has reported the podoplanin and CD44 double positive cells possess cancer stem cell properties in A431 cell line [46]. We therefore used these two markers to examine the effect of KLF4 knockdown on this stem cell population. As expected, the proportion of podoplanin+/CD44+ cells reduced from 41.7% in control cells (siCon cells) to 22% in KLF4 knockdown cells (siKLF4 cells) (Figure 6C). Our mouse studies have shown that KLF4 was important for wound healing, a process that involves keratinocyte proliferation and migration. To test the effect of KLF4 on cell migration *in vitro,* we used KLF4 knockdown A431 cells and performed a scratch assay. 2×10^6^ cells were seeded into 6-well plates and cultured to full confluence and then applied to this assay. At 12 h after the scratch, the percentage of wound closure for siCon cells was 40%, whereas siKLF4 cells only showed 12% closure, indicating that KLF4 was essential for cell migration in A431 cells (Figure 6D, E). Consistently, overexpression of KLF4 in A431 cells increased cell migration accompanied by increased cancer stem cell population by flow cytometric analysis using podoplanin and CD 44 antibodies (Figure S4 and data not shown).

## Discussion

KLF4 function in terminally differentiated epithelial cells of skin includes regulation of the cell cycle, proliferation and differentiation [47]. However, KLF4 expression and potential function in epidermal stem cells has not been studied before. Here we utilized 6–8 week-old mice when hair follicles are in the resting (telogen) phase in which follicles lie dormant without significant proliferation, apoptosis or differentiation. Using the KLF4/EGFP mouse model, we found that KLF4 was expressed in CD34+/CD49f+ bulge stem cell-enriched populations (Figure 1D). The quiescent nature of KLF4-expressing cells was also shown by co-localization of KLF4 expressing cells with BrdU label retaining cells (Figure 2A, B). We noticed that KLF4 gene expression in CD34+/CD49f+/Lrig1+ cells was about 2.2 fold higher than in CD34+/CD49f-/Lrig- cells sorted from wild-type mice. High levels of KLF4 expression in most differentiated, post mitotic skin epithelial cells [48] and low percentage of skin epidermal stem cells may be a reason why a bigger difference has not been observed. Nevertheless, our studies collectively provide the first evidence that KLF4 was likely expressed in mouse hair follicle stem cells, especially in bulge stem cells.

We found that the proportion of KLF4-positive cells in LRCs was 4.1%, suggesting that only a subset of these LRCs expressed KLF4. These results reveal a heterogeneous nature of LRCs. Different nature of KLF4-expressing and KLF4-non-expressing LRCs and the related functional influence in wound healing are currently unknown. Our studies also showed that KLF4 knockout decreased the population of CD34+/CD49f+ cells accompanied by reduced self-renewal ability of these cells. Together with the label retaining ability of KLF4 expressing cells, our results indicated KLF4 plays an important role in the homeostasis of skin bulge stem cells. In addition, expression of KLF4 in rare skin stem cells and in the bulk of differentiated keratinocytes may suggest that the functions of KLF4 in these populations are different. Alternatively but not mutually exclusively, different KLF4 isoforms may exist to deliver different functions as recently reported in pancreatic cancer cells [49]. Characterization of different KLF4 isoforms and/or separation of distinct KLF4 expressing cells will be necessary for dissecting specific functions of KLF4 in skin homeostasis as well as pathogenesis including wound healing.

Previous work has demonstrated that stem cells located in the bulge area [12] and isthmus [50] contributed to wound healing. Here we showed that KLF4-expressing multipotent cells participated in re-epithelialization during cutaneous wound healing. It is known that cutaneous wounds still heal in the absence of hair follicle stem cell contribution at the cost of delayed reepithelialization [51]. From our study, KLF4 expression in possible hair follicle stem cells may contribute to the wound healing (Figure 5). We also noticed that KLF4-expressing stem cells remained quiescent as evidenced by rarely detectable blue cells eight months after the cells were labeled (Figure S3E). However, they were readily activated and detectable by wound placement (Figure S3F). This observation is consistent with a recent proposal for olfactory neural stem cells, stem cells within the LRC population, when present, may represent a reserve population activated after tissue damage, whereas under normal conditions, a cycling population of stem cells maintains homeostasis[52]. Moreover, KLF4 deficiency delayed the process of wound healing and cell migration. It has been reported that KLF4 is required for establishing skin barrier function as evidenced by KLF4 deficiency selectively perturbing the differentiation of the late-stage structures such as cornified envelope ENREF[1]. It is not clear whether the role of KLF4 in barrier function is also involved in wound healing in our setting. Finally, our wound healing model did not limit for contraction. Although this simple method allowed us to observe an obvious phenotype, more rigorous models should be used in the future in order to define the role of KLF4 in the complex wound healing process. For example, an early inflammation model [53] and other surgical models including pressure ulcer model and mechanical load model with limit for contraction could be used [54]. Nonetheless, our results suggest a critical function of KLF4-expressing epidermal multipotent stem cells in cutaneous wound healing.

## Supporting Information

Figure S1 Generation of KLF4/EGFP and KLF4/CreER™ mouse models.
(PDF)

Figure S2 Quiescent nature of KLF4 expressing cells around mouse hair follicles.
(PDF)

Figure S3 KLF4-expressing pluripotent cells contributed to cutaneous wound healing.
(PDF)

Figure S4 KLF4 overexpressing A431 cells showed increased cell migration.
(PDF)
