# Supplementary material for: Expression of Kruppel-Like Factor KLF4 in Mouse Hair Follicle Stem Cells Contributes to Cutaneous Wound Healing
Source: PLoS One. 2012 Jun 20;7(6):e39663. doi: 10.1371/journal.pone.0039663 (PMC3379995; doi:10.1371/journal.pone.0039663)
Supplement: Figure S1 — Generation of KLF4/EGFP and KLF4/CreER™ mouse models. (PDF) [file pone.0039663.s001.pdf]

Figure S1

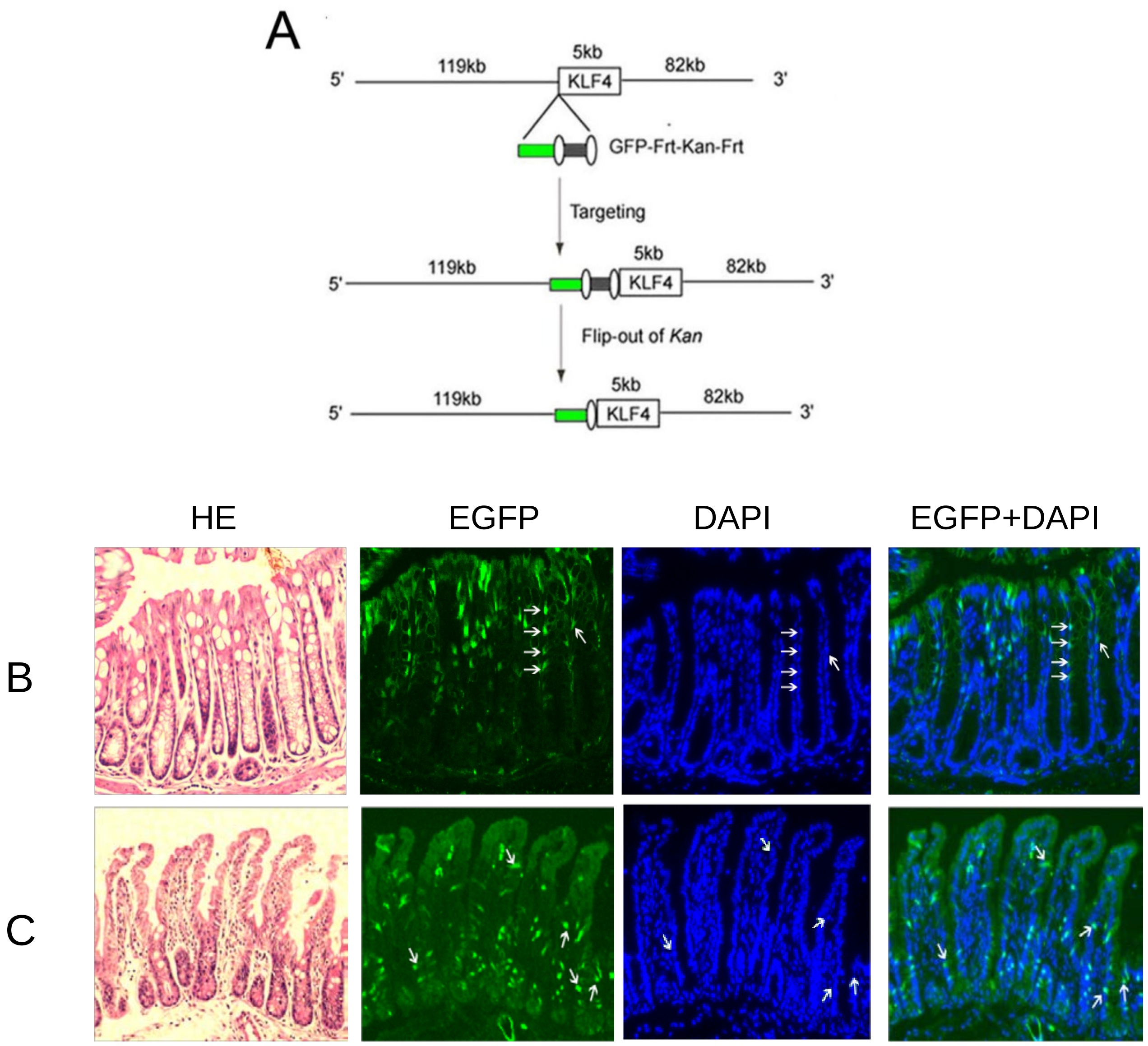

Figure S1. Generation of KLF4/EGFP and KLF4/CreER™ mouse models. (A) A targeting strategy to generate KLF4/EGFP transgene. A PCR generated fragment, consisting of EGFP cDNA (green box) and Kanamycin resistant gene cassette (gray box) between two Frt fragments (ellipses), was inserted into a mouse KLF4 gene (bigger rectangle)-containing BAC clone at the position of KLF4 translation start site by homologous recombination. KLF4/CreER™ transgene was similarly generated. (B) and (C) showed KLF4 expression patterns in mouse colon and small intestine respectively under direct a fluorescent microscope with staining. These patterns matched those of previously published ones. Note that in the small intestine, KLF4/EGFP mainly showed diffused cytoplasmic expression in villi with strong signals seen in goblet cells in villi and crypts. The matched expression pattern of Cre recombinase from KLF4/CreER™ transgenic mice with that of endogenous KLF4 was also observed by immnohistochemical staining of Cre (data not shown).
