# Supplementary material for: Expression of Kruppel-Like Factor KLF4 in Mouse Hair Follicle Stem Cells Contributes to Cutaneous Wound Healing
Source: PLoS One. 2012 Jun 20;7(6):e39663. doi: 10.1371/journal.pone.0039663 (PMC3379995; doi:10.1371/journal.pone.0039663)
Supplement: Figure S2 — Quiescent nature of KLF4 expressing cells around mouse hair follicles. (PDF) [file pone.0039663.s002.pdf]

Figure S2

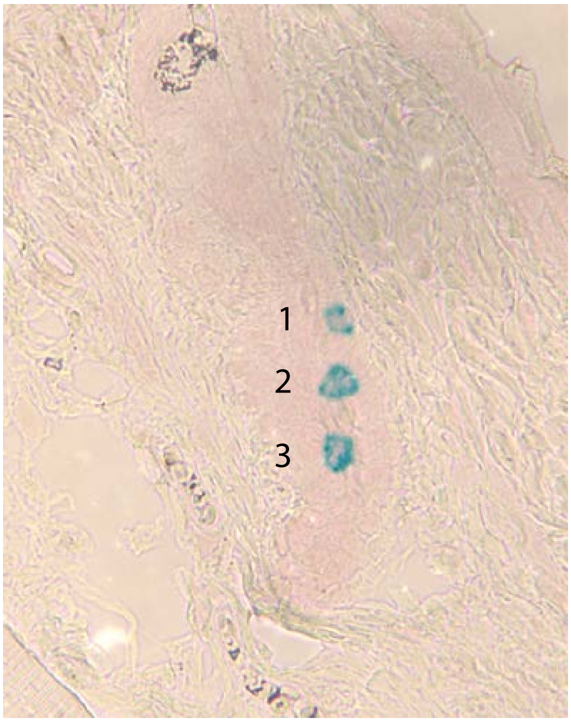

Figure S2: Quiescent nature of KLF4 expressing cells around mouse hair follicles. 5-week-old KLF4/CreER™(+/-)/Rosa26RLacZ mice were induced by intraperitoneal injection tamoxifen (100 mg/kg) daily for 5 consecutive days. Twenty eight days later, mice were sacrificed and frozen slides were made from skin tissues, followed by X-gal staining to trace KLF4-expressing cells. Note that 4 possible cells were present in the blue cluster 2, suggesting a slow cycling nature of these hair follicle cells.
