# Supplementary material for: Expression of Kruppel-Like Factor KLF4 in Mouse Hair Follicle Stem Cells Contributes to Cutaneous Wound Healing
Source: PLoS One. 2012 Jun 20;7(6):e39663. doi: 10.1371/journal.pone.0039663 (PMC3379995; doi:10.1371/journal.pone.0039663)
Supplement: Figure S3 — KLF4-expressing pluripotent cells contributed to cutaneous wound healing. (PDF) [file pone.0039663.s003.pdf]

Figure S3

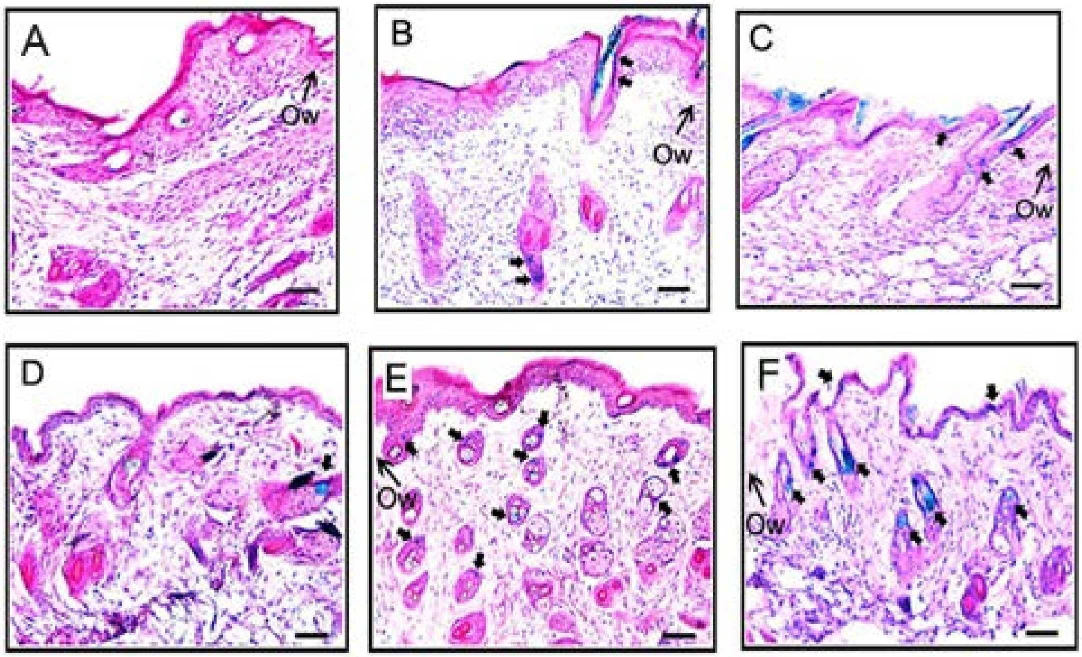

Figure S3. KLF4-expressing pluripotent cells contributed to cutaneous wound healing. Wounds were introduced into the backs of KLF4/CreER™/Rosa26RLacZ mice after 3 months of control (A) or tamoxifen (B and C) induction. Migration of KLF4 expressing pluripotent cells towards the wound area was detected by X-gal staining. (D-F) Similar to A-C except that wound was placed eight months after induction. X-gal staining was performed in intact skin tissues (D) and upon wound placements (E and F). Results shown are representative of three independent experiments. Ow, open wound area. Arrows indicated positive blue staining. Scale bars, 50 µm.
