# Supplementary material for: Expression of Kruppel-Like Factor KLF4 in Mouse Hair Follicle Stem Cells Contributes to Cutaneous Wound Healing
Source: PLoS One. 2012 Jun 20;7(6):e39663. doi: 10.1371/journal.pone.0039663 (PMC3379995; doi:10.1371/journal.pone.0039663)
Supplement: Figure S4 — KLF4 overexpressing A431 cells showed increased cell migration. (PDF) [file pone.0039663.s004.pdf]

Figure S4

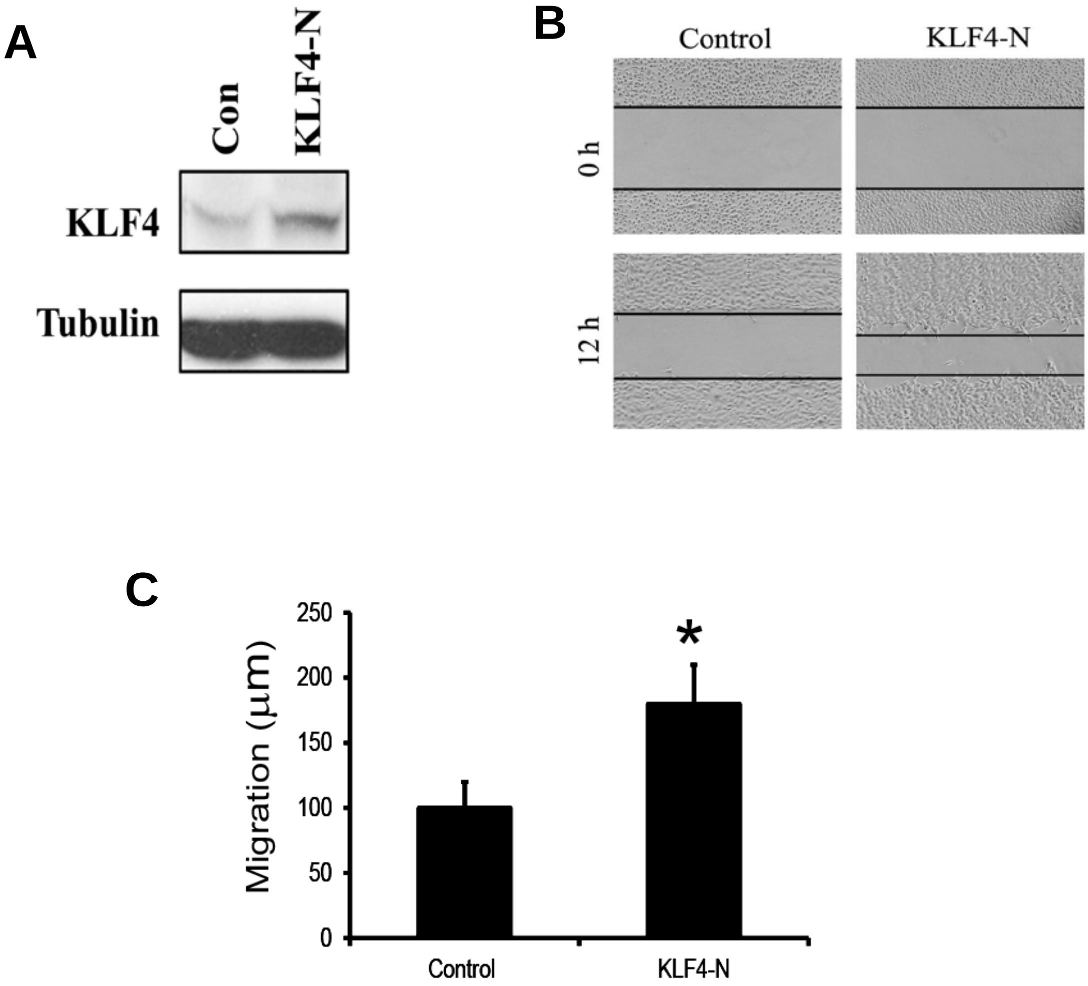

Figure S4: KLF4 overexpressing A431 cells showed increased cell migration. (A) KLF4 expression was examined in control (Con) and KLF4 overexpressed (KLF4-N) cells by Western Blotting analysis. (B). Scratch assays were performed using Con and KLF4-N cells. 2×10^6 cells were cultured to confluence, then scratched and photographed immediately (0 h), or after 12 hours (12 h). C. Quantitation of cell migration at 12h after scratching. Error bars represent standard error from three separate experiments. *P<0.05 vs control.
